# Environmental Lead: Rabito et al. Respond

**DOI:** 10.1289/ehp.1104909R

**Published:** 2012-05-01

**Authors:** Felicia A. Rabito, Janet C. Rice, Whitney Arroyave

**Affiliations:** School of Public Health and Tropical Medicine, Tulane University, New Orleans, Louisiana, E-mail: rabito@tulane.edu

We thank Mielke for his interest in our study of residential lead hazards in New Orleans (Rabito et al. 2012). In our study we found that most homes had either dust or soil lead above federal standards and concluded that children are at risk for lead exposure. Regarding the specific issue of whether soil lead has increased or decreased, we agree that a direct comparison between census-level soil lead survey data and direct measure-ments of residential samples has limitations. To reiterate the limitations outlined in our article (Rabito et al. 2012), differences in the number of soil samples, the sampling methodology, and the sample location limited our ability to directly measure any change in soil lead levels.

Although it is of interest to know whether soil lead has increased or decreased as a result of Hurricane Katrina, our focus was to measure current residential lead hazards, because lead contamination in and around homes is the primary pathway of exposure for children (Lanphear and Roghmann 1997). Our finding that 61% of homes had lead levels above federal standards provides evidence that New Orleans children are at high risk of exposure. That the samples were collected from homes typically considered to be low risk (given the socio-demographic characteristics of the sample) is cause for added concern.

In his letter, Mielke refers to trends in blood lead levels. The issue of whether blood lead levels are increasing or decreasing in New Orleans children—which was not within the scope of our study (Rabito et al. 2012)—is not easily addressed using surveillance data. Screening practices and rates drive the reported prevalence in any given year. Zahran et al. (2010) compared aggregate blood lead levels derived from the Louisiana Childhood Blood Lead Surveillance System (CBLSS) pre-Hurricane Katrina to levels post-Hurricane Katrina and found that median blood lead had decreased. However, profound changes in the socio-demographic makeup of the New Orleans population make the comparison vulnerable to significant bias (Bloomberg 2011). The surveillance data provided by Mielke supports our notion that the post-Hurricane Katrina population screened under the CBLSS is not stable from year to year. As stated by Mielke, 6.4% of children had elevated blood lead levels in 2008, but the estimate decreased to 3.3% in 2010. This decrease might not be valid because the majority of children in the 2008 cohort were from the inner city (with the highest prevalence of old housing) whereas the 2010 cohort included a higher proportion from outer areas of the city (considered low risk based on housing age). The assumption by Zahran et al. (2010) that soil lead serves as a proxy for length of lead exposure is likely not valid given the amount of housing destabilization and subsequent mobility of the population in the years following the storm. Finally, low screening rates, coupled with a reporting level of > 10 µg/dL (widely accepted as above the level of concern) does not allow for the valid estimation of the preva-lence of elevated blood lead levels in New Orleans children.

Regardless of the inconclusive nature of blood lead data, given that 61% of sampled homes have significant lead hazards, we maintain that New Orleans children who live in old housing are at risk for lead exposure independent of race or income. We support the recent statement by the Centers for Disease Control and Prevention Advisory Committee on Childhood Lead Poisoning Prevention that “the goal of primary prevention is to ensure that all homes become lead-safe and do not contribute to childhood lead exposure” (Centers for Disease Control and Prevention 2012). The City of New Orleans has made strides in its fight to reduce lead hazards in public places; however, they should not relax in their efforts to protect the environ-ment from further contamination.
